# Noninvasive imaging-based machine learning algorithm to identify progressive disease in advanced hepatocellular carcinoma receiving second-line systemic therapy

**DOI:** 10.1038/s41598-023-37862-y

**Published:** 2023-07-01

**Authors:** Wei Dong, Ye Ji, Shan Pi, Qi-Feng Chen

**Affiliations:** 1 Department of Medical Oncology, Nanyang Second Peopleʹs Hospital, Nanyang, China; 2 Department of Medical Oncology, Nanyang Central Hospital, Nanyang, China; 3 Department of Radiology, The Third Affiliated Hospital, Sun Yat-Sen University, No. 600 Tianhe Road, Guangzhou, 510630 Guangdong China; 4 Department of Medical Imaging and Interventional Radiology, Sun Yat-Sen University Cancer Center, 651 Dongfeng Road East, Guangzhou, 510060 Guangdong China; 5 State Key Laboratory of Oncology in South China, Guangzhou, Guangdong China; 6 Collaborative Innovation Center for Cancer Medicine, Guangzhou, Guangdong China

**Keywords:** Cancer, Cancer imaging, Gastrointestinal cancer, Immunology, Immunotherapy, Tumour immunology, Cancer imaging

## Abstract

The aim of this study was to predict tyrosine kinase inhibitors (TKI) plus anti-PD-1 antibodies (TKI-PD-1) efficacy as second-line treatment in advanced hepatocellular carcinoma (HCC) using radiomics analysis. From November 2018 to November 2019, a total of 55 patients were included. Radiomic features were obtained from the CT images before treatment and filtered using intraclass correlation coefficients (ICCs) and least absolute shrinkage and selection operator (LASSO) methods. Subsequently, ten prediction algorithms were developed and validated based on radiomic characteristics. The accuracy of the constructed model was measured through area under the receiver operating characteristic curve (AUC) analysis; survival analysis was performed via Kaplan–Meier and Cox regression analyses. Overall, 18 (32.7%) out of 55 patients had progressive disease. Through ICCs and LASSO, ten radiomic features were entered into the algorithm construction and validation. Ten machine learning algorithms showed different accuracies, with the support vector machine (SVM) model having the highest AUC value of 0.933 in the training cohort and 0.792 in the testing cohort. The radiomic features were associated with overall survival. In conclsion, the SVM algorithm is a useful method to predict TKI-PD-1 efficacy in patients with advanced HCC using images taken prior to treatment.

## Introduction

Hepatocellular carcinoma (HCC) was the sixth most prevalent cancer and the fourth leading cause of cancer death worldwide in 2018^1^. HCC imposes a heavy disease burden in developing countries, especially in Eastern Asia and sub-Saharan Africa, with a majority of patients suffering from advanced disease^2^.

Systematic treatments, including tyrosine kinase inhibitors (TKIs) and immune checkpoint inhibitors (ICIs), are recommended for patients with advanced HCC. Prior to 2020, sorafenib^3,4^ and lenvatinib^5^ were the primary first-line treatments for advanced HCC. However, in 2020, the publication of the IMbrave 150 trial demonstrated that the combination of atezolizumab and bevacizumab (T + A) resulted in superior outcomes to sorafenib^6^. Additionally, in 2022, the combination of tremelimumab and durvalumab (D + T) also proved more effective than sorafenib^7^. As such, T + A and D + T are now the preferred first-line treatments for advanced HCC, while sorafenib, lenvatinib, durvalumab, and pembrolizumab are other recommended regimens according to NCCN guidelines^8^. However, for patients who have failed first-line therapy, there is no established optimal course of treatment under NCCN guidelines^8^. The Barcelona Clinic Liver Cancer (BCLC) guidelines still regard treatment after first-line failure as an area of ongoing research^9^. Between July 2018 and July 2021, Lei et al. conducted a study involving patients with unresectable HCC (uHCC) who had failed sorafenib treatment^10^. They discovered that a combination of TKI and PD-1 inhibitors (TKI-PD-1) was more advantageous than TKI monotherapy for these patients, indicating that TKI-PD-1 therapy could be a promising treatment option for advanced HCC after first-line failure.

However, a subset of patients still progress during TKI-PD-1 therapy, and thus the identification of biomarkers to predict each patient’s response to combination therapy is essential. To date, serum and tissue sample analyses have not clearly defined the subpopulation most likely to progress^2^. In addition to collecting invasive serum and tissue samples, noninvasive imaging is routinely performed during routine medical examinations. Radiomics comprise valuable information sources for prognostication^11^. Machine learning can automatically construct models to interpret medical images based on their radiomic features dataset. A predictive approach using CT-based radiomics is a noninvasive, cost-effective way to identify patients at high risk of PD during TKI-PD-1 therapy. However, such machine learning methods need further study before applying radiomics in the clinic. Therefore, this study was carried out to construct and compare machine learning models based on radiomics for predicting the response of advanced HCC to combined TKI-PD-1 treatment as second-line treatment.

## Results

### Patient population

**Figure 1:**
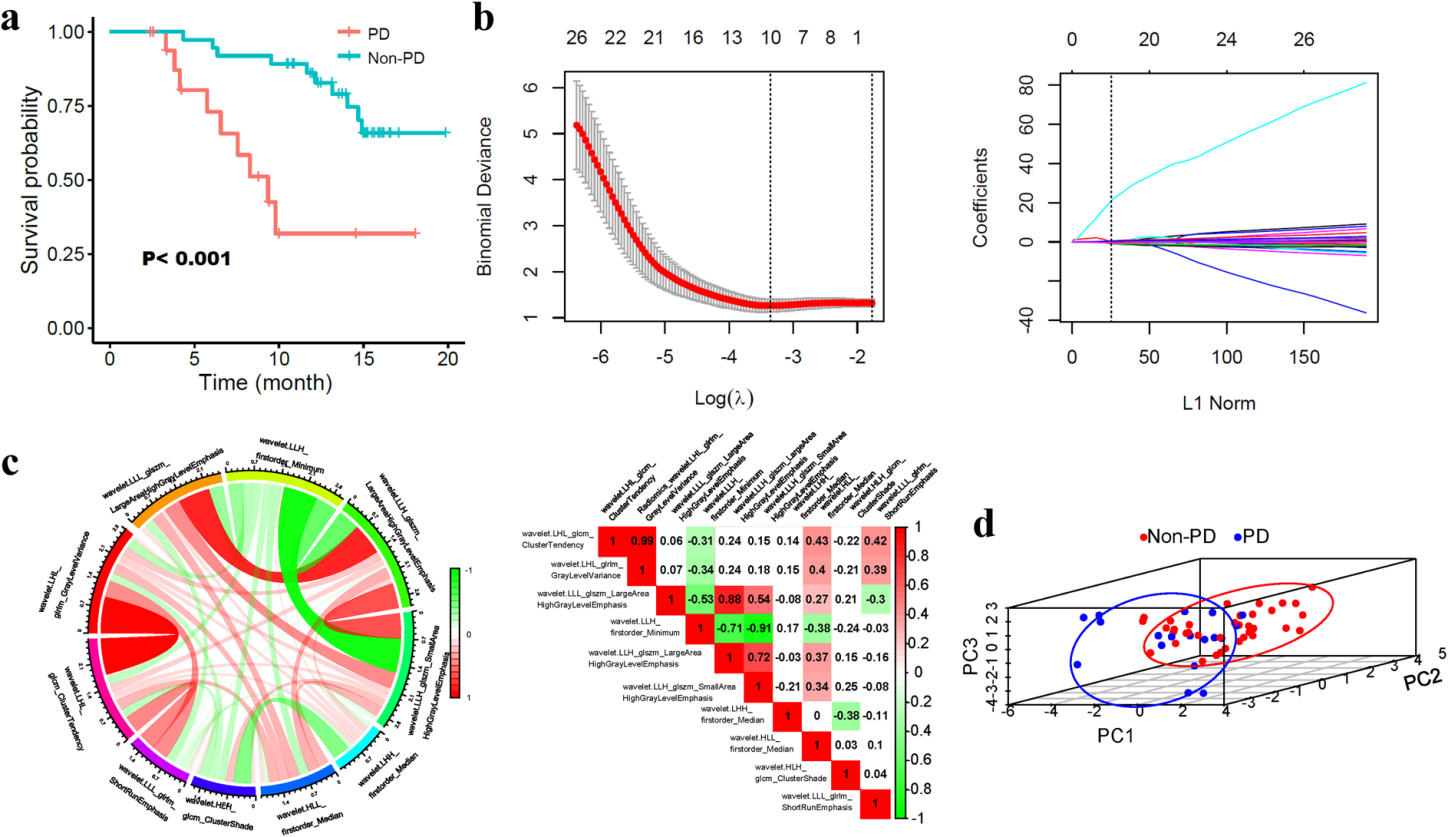
Radiomics features of the selection process for predicting progressive disease (PD). (**a**) PD and non-PD stratified the overall survival (OS) of HCC patients treated with TKI-PD-1. (**b**) LASSO method to find the 10 most meaningful features of PD. (**c**) A chord diagram and heatmap illustrate the correlation matrix among all 10 features. In the left chord diagram, the wider the bands are, the closer the correlation. In the right heatmap (generated by R software version 3.6.0), correlated paired features are exhibited with color backgrounds. Red indicates a positive correlation; green indicates a negative correlation. (**d**) Principal component analysis of the features correlated with PD.

A total of 55 patients were enrolled in the study, including 37 (67.3%) patients with non-progression disease (non-PD) and 18 (32.7%) patients with PD. The PD patients experienced a shorter overall survival (OS) than the non-PD patients (9.35 months versus not reached, *P* < 0.001, Fig. 1a). The median patient age in the overall setting was 53.0 years (range, 43.0–55.0), and most patients were male (90.9%). The etiology of hepatitis B HCC accounted for 94.5%, and 67.3% of patients had an alpha fetoprotein level > 200 ng/mL. Their mean albumin-bilirubin (ALBI) score was − 2.60, which was relatively higher in PD patients than in non-PD patients (− 2.29 versus − 2.76, *P* < 0.001). Median platelet (PLT), aspartate transaminase (AST), alanine transaminase (ALT), and alkaline phosphatase (ALP) levels were 194.00*10^9^/L, 60.30 U/L, 44.50 U/L, and 134.10 U/L, respectively. At baseline, 61.8% and 38.2% of patients had multiple tumors and a single nodule, respectively. The tumor diameter was > 5 cm in 81.8% of patients and < 5 cm in 18.2%. The proportions of patients with macroscopic vascular invasion and extrahepatic spread at baseline were 80% and 34.5%, respectively. Characteristic differences were not reported for these variables (*P* > 0.05) except for ALBI (Table 1).

**Table 1:** Patients’ demographic characteristics.

	Overall (n = 55)	PD (progressive disease, n = 18)	Non-PD (n = 37)	*P*-value
Sex (%)				0.160
Female	5 (9.1)	0 (0.0)	5 (13.5)	
Male	50 (90.9)	18 (100.0)	32 (86.5)	
Age (median [interquartile range, IQR])	53.00 [43.00, 55.00]	51.50 [48.50, 55.00]	53.00 [43.00, 57.00]	0.336
Hepatitis B virus infection (%)				0.247
No	3 (5.5)	2 (11.1)	1 (2.7)	
Yes	52 (94.5)	16 (88.9)	36 (97.3)	
Platelets (median [IQR])	194.00 [162.50, 282.50]	198.50 [160.50, 319.00]	192.00 [164.00, 281.00]	0.851
Albumin-bilirubin (mean (standard deviation, SD))	− 2.60 (0.44)	− 2.29 (0.38)	− 2.76 (0.38)	< 0.001
Aspartate aminotransferase (median [IQR])	60.30 [36.90, 95.00]	64.85 [40.17, 116.58]	60.30 [36.90, 84.10]	0.379
Alanine transaminase (median [IQR])	44.50 [30.45, 66.30]	48.75 [28.85, 71.20]	40.90 [30.70, 66.00]	0.713
Alkaline phosphatase (median [IQR])	134.10 [102.90, 182.50]	137.75 [101.62, 219.65]	131.00 [106.60, 173.60]	0.446
Alpha fetoprotein level (%)				0.761
< 200 ng/ml	18 (32.7)	5 (27.8)	13 (35.1)	
≥ 200 ng/ml	37 (67.3)	13 (72.2)	24 (64.9)	
Number (%)				> 0.99
Single	21 (38.2)	7 (38.9)	14 (37.8)	
Multiple	34 (61.8)	11 (61.1)	23 (62.2)	
Diameter (%)				0.713
< 5 cm	10 (18.2)	4 (22.2)	6 (16.2)	
≥ 5 cm	45 (81.8)	14 (77.8)	31 (83.8)	
Vascular invasion (%)				> 0.99
No	11 (20.0)	3 (16.7)	8 (21.6)	
Yes	44 (80.0)	15 (83.3)	29 (78.4)	
Distant spread (%)				0.764
No	36 (65.5)	11 (61.1)	25 (67.6)	
Yes	19 (34.5)	7 (38.9)	12 (32.4)	

### Radiomics feature selection and analysis

In total, 2,458 features were acquired for every patient, among which 568 features were excluded with intraclass correlation coefficients (ICCs) < 0.90, leaving 1,890 features for further analysis. Then, 10 discriminatory wavelet-related features between the non-PD and PD patients assessed by RECIST 1.1 were identified via least absolute shrinkage and selection operator (LASSO) feature selection (Fig. 1b, Supplementary Table S1).

After obtaining these 10 features, we further explored their relationships using Spearman correlation analysis. The Chord diagram shows links between the features (shown in Fig. 1c). Obviously, warm red wide bands indicated feature 1 and feature 2, feature 3 and feature 5, and feature 5 and feature 6 were significantly positively related to each other in the network. Meanwhile, features 4 and 6 were negatively related to the green wide band connection. Overall, the detailed relationships among features are shown in heatmap Fig. 1c, and feature 1 and feature 2 had the highest correlation coefficient (0.99) in Spearman analysis. We next performed a principal component analysis (PCA) process to compress the features and reduce the dimensionality. As visualized in Fig. 1d, the PD patients (left bottom cluster) could be separated from the non-PD patients (right top cluster).

Differential values were distributed between PD and non-PD patients for all radiomics features (Supplementary Figure S1). Higher expression was noted for features 1, 2, 4, 7, and 10 in non-PD patients, and the difference for feature 1 was significant (*P* < 0.05). In contrast, PD patients showed relatively high expression of features 3, 5, 6, 8, and 9, but the difference was not significant (*P* > 0.05). The area under the receiver operating characteristic curve (AUC) for the prediction of PD ranged from 0.572 to 0.664 for the ten features, as shown in Supplementary Figure S2. Among these features, the AUC of feature 4 was 0.661 (95% CI: 0.500–0.822) and the AUC of 6 was 0.664 (95% CI: 0.509- 0.818), both of which reached significance in ROC analysis (*P* < 0.05).

### Radiomics-based machine learning algorithm predicts the response

**Figure 2:**
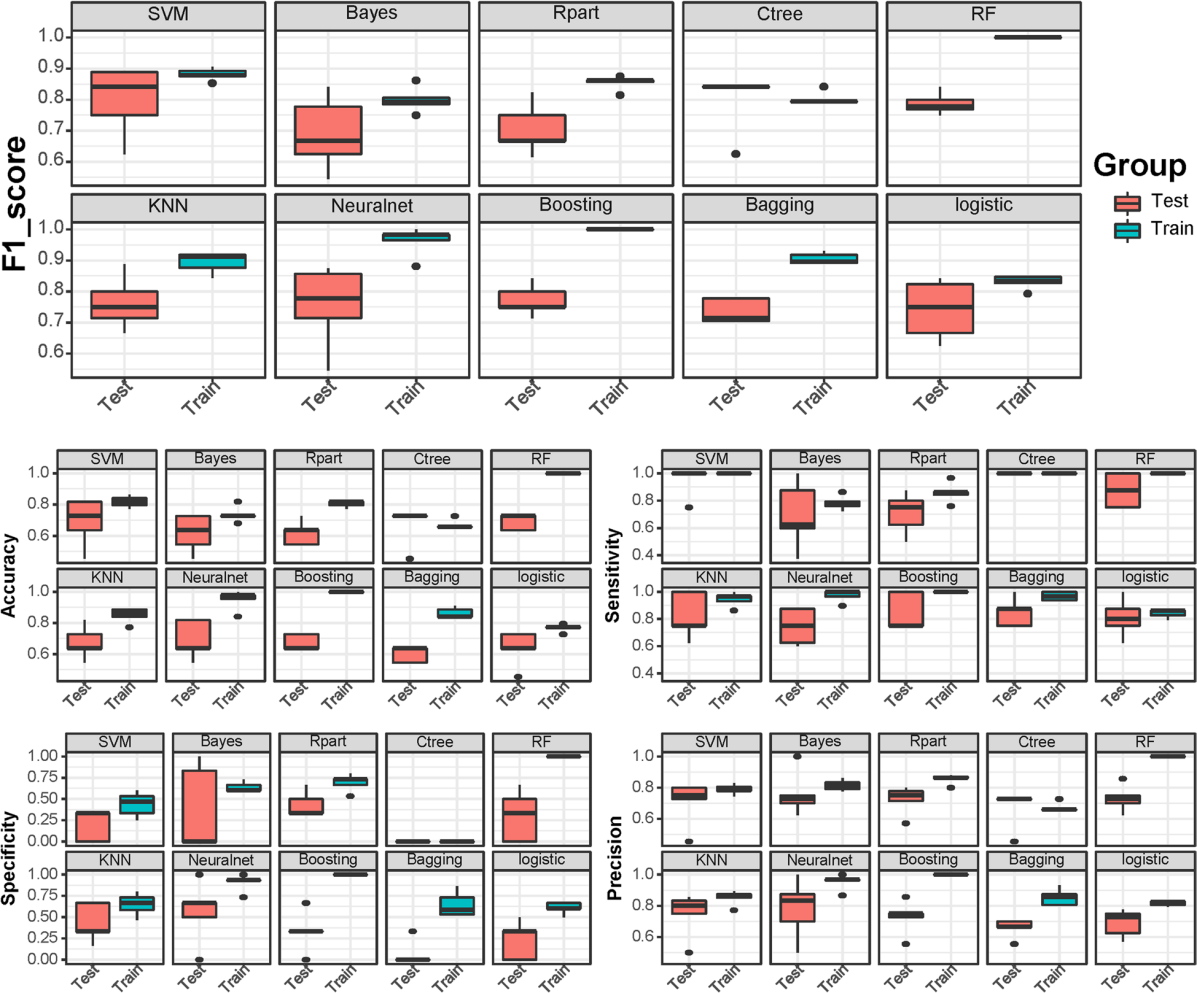
The box plot shows the F1-score, accuracy, sensitivity, specificity, and precision of ten machine learning models in the training and testing cohorts.

Ten algorithms distinguished PD from non-PD disease with varied efficacy. Overall, better prediction results were observed in the training cohort than in the test cohort (Fig. 2). All ten algorithms, including support vector machine (SVM), naïve Bayes (NB), recursive partitioning and regression trees (Rpart), conditional inference trees (Ctree), random forests (RF), k-nearest neighbors (KNN), neuralnet, boosting, bagging, and logistics, obtained mean F1 scores of 0.88, 0.80, 0.85, 0.80, 1.00, 0.89, 0.96, 1.00, 0.91, and 0.82 in the training cohort and 0.80, 0.69, 0.70, 0.80, 0.79, 0.76, 0.75, 0.77, 0.74, and 0.74 in the testing cohort, respectively. Overall, the classification model using the SVM algorithm achieved the best F1 score. Moreover, the SVM algorithm predicted non-PD and PD with a mean accuracy, sensitivity, specificity and precision of 81.8%, 100.0%, 43.7%, and 78.8%, respectively, in the training cohort and 69.1%, 95.0%, 20.0%, and 70.6%, respectively, in the testing cohort (Supplementary Table S2).

**Figure 3:**
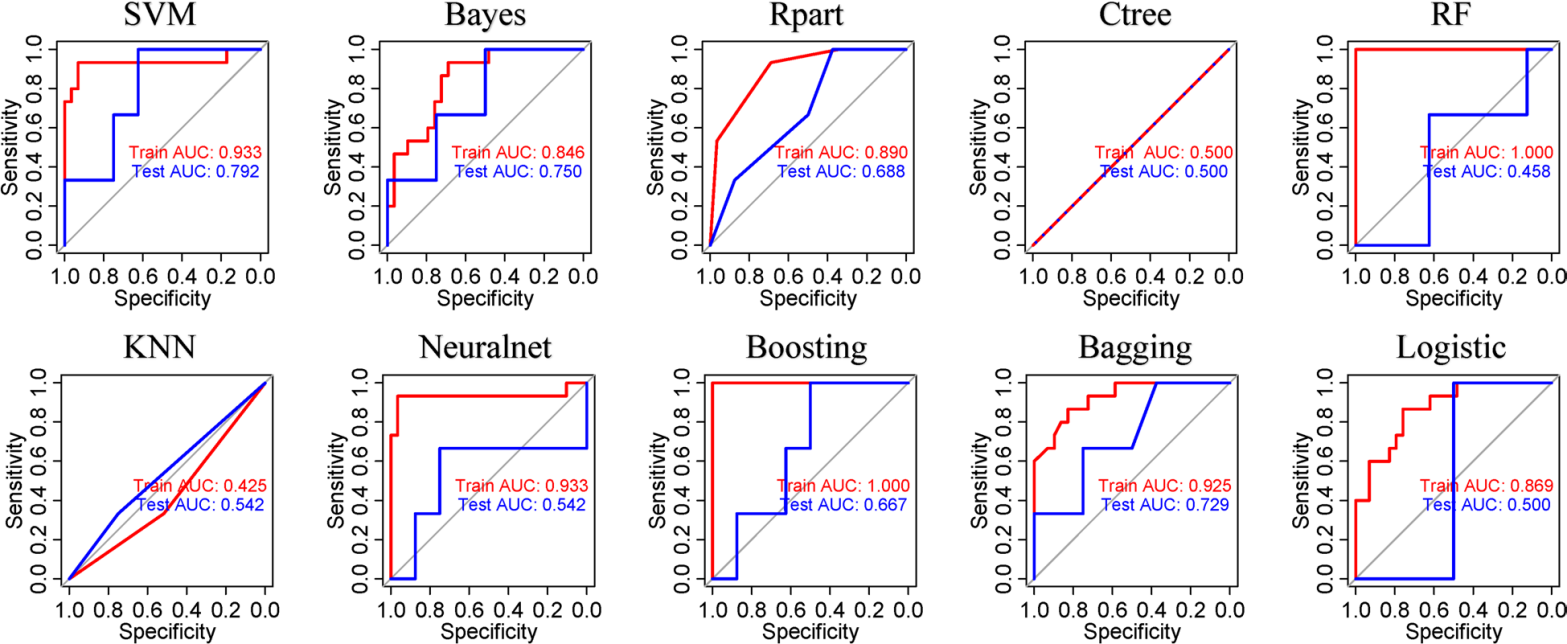
The overall accuracy of ten machine learning algorithms was assessed by area under the receiver operating characteristic curves in the training and testing cohorts.

The AUC values of each model were generally higher in the training cohort than in the test cohort (Fig. 3). Different accuracies of the models were exhibited, among which the SVM model had the highest AUC score of 0.933 in the training cohort and 0.792 in the testing cohort. The SVM, Bayes, and bagging algorithms all achieved AUCs > 0.7 in the test cohort.

### Survival analysis

**Figure 4:**
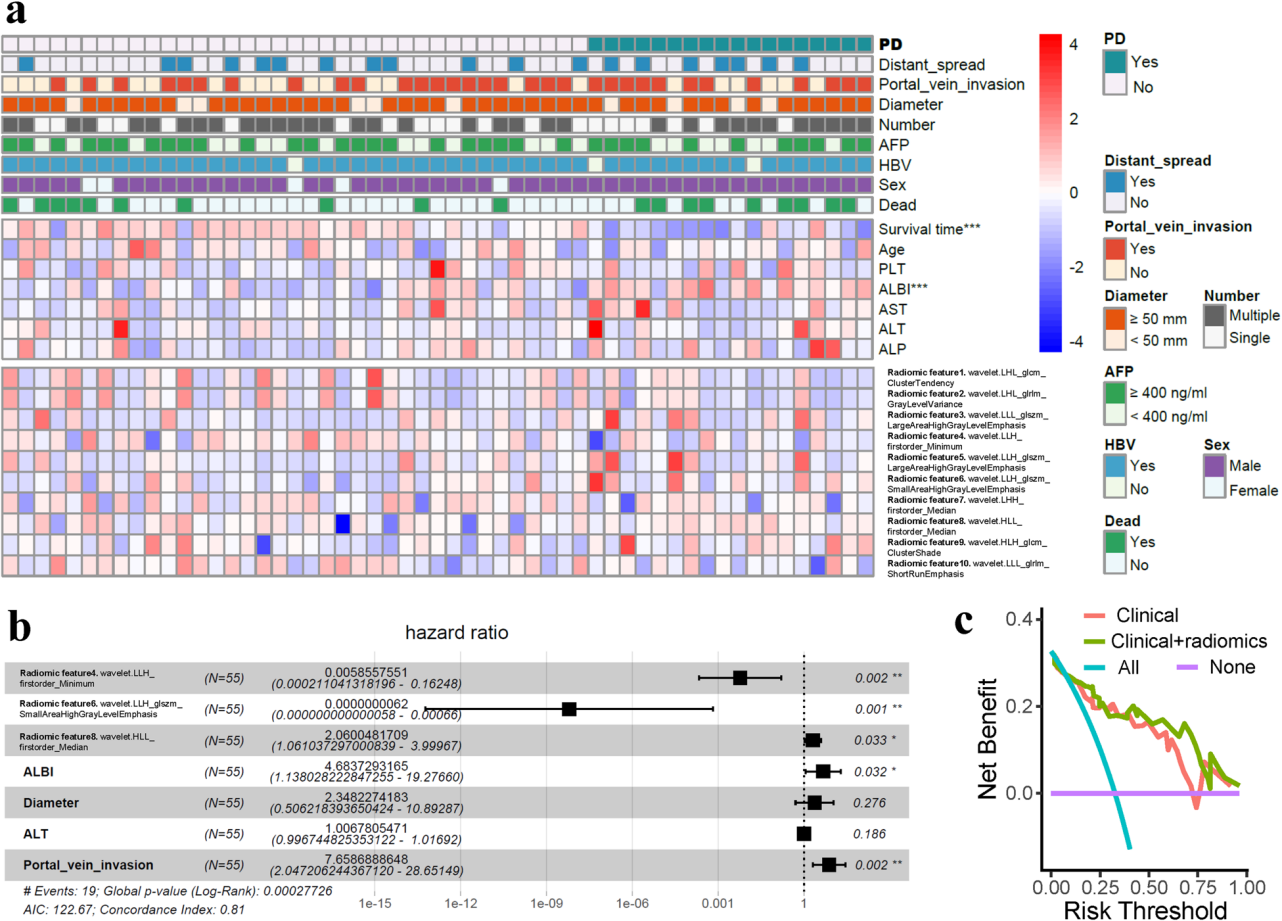
The landscape of the radiomic features and clinical characteristics. (**a**) The progressive disease (PD) patients had shorter survival times and higher albumin-bilirubin (ALBI) scores; their radiomic features exhibited differential expression levels between PD and non-PD patients. (**b**) Radiomic features 4, 6, and 8, ALBI, and portal vein invasion were survival-associated factors in univariable and multivariable Cox regression survival analysis. (**c**) Additional net benefit of radiomics in decision curve analysis. ***P < 0.001; **P < 0.01; *P < 0.05.

Overall, 19 patients died during the follow-up. OS was not reached in all patients, and the OS in subgroups based on the drugs used is presented in Supplementary Figure S3, which shows no significant difference among subgroups according to the drugs used (*P* = 0.990). The log-rank test was used to compare high versus low radiomic features with survival. Four out of ten features were found to be significantly associated with OS, including feature 4 (*P* = 0.039), feature 5 (*P* = 0.049), feature 8 (*P* = 0.018), and feature 9 (*P* = 0.041), as shown in Supplementary Figure S4. Cox regression analysis incorporating radiomic features and clinical variables was conducted to identify survival-associated factors (Fig. 4a). A total of 7 factors (features 4, 6, 8, ALBI, tumor diameter, ALT, and portal vein invasion) generated in the univariate Cox regression model were further selected to be included in the multivariate Cox regression model. Finally, feature 4 (*P* = 0.002) and feature 6 (*P* = 0.001) were favorable survival factors, while feature 8 (*P* = 0.033), ALBI (*P* = 0.032), and portal vein invasion (*P* = 0.002) were hazardous survival factors in the multivariate Cox regression model with a c-index of 0.81 (Fig. 4b). Figure 4c shows the more net benefit of using a model comprising clinical characteristics plus radiomics than using a clinical model.

## Discussion

The present study demonstrated that radiomic features extracted from initial CT images before TKI-PD-1 treatment could predict the HCC treatment response. The radiomics-based SVM machine learning algorithm using ten LASSO filtered features predicted the PD response with a mean accuracy, sensitivity, specificity, precision, and F1 score of 81.8%, 100.0%, 43.7%, 78.8%, and 88.0%, respectively, in the training cohort and 69.1%, 95.0%, 20.0%, 70.6%, and 80.0%, respectively, in the testing cohort.

Treatment options for advanced HCC have rapidly evolved over the past several years. The optimal course of treatment for advanced HCC patients who do not respond to first-line therapy is currently not well established^12,13^. Since TKIs target vascular endothelial growth factor receptors (VEGFRs) and modulate the immune microenvironment, the treatment efficacy of PD-1 inhibitors would be theoretically improved when combined with TKIs^14^. Indeed, emerging clinical data suggest that although a single agent has demonstrated disease control with manageable toxicity, improved synergistic outcomes have been shown. Lenvatinib combined with pembrolizumab resulted in a DCR of > 85% in a recent phase Ib study^2^. In a phase II trial RESCUE, the DCR was > 77% in the first-line and 75% in the second-line advanced HCC cohort treated with camrelizumab combined with apatinib^15^. Additionally, a recent study demonstrated that a combination of TKI-PD-1 was found to be more advantageous than TKI monotherapy for HCC patients who had previously failed sorafenib treatment^10^. This suggests that TKI-PD-1 therapy could be a promising treatment option for advanced HCC after first-line therapy failure.

The additional refinement of the subpopulation of patients most likely to benefit from this combination therapy would be of clinical significance. However, reliable prediction tools to support the precise therapy of TKI-PD-1 are not currently available and are urgently needed in the era of immunotherapy. To date, predictive markers of response or resistance using serum or tissue samples as biomarkers for advanced HCC have not been clearly defined, including PD-L1 expression and tumor mutation burden (TMB) levels^16,17^. Recently, Yang et al. found that copy number variations (CNVs) in plasma cell-free DNA (cfDNA) could predict the clinical result of combined PD-1 inhibitor and lenvatinib therapy and other immune checkpoint inhibitor-based therapies in hepatobiliary cancers^18^. However, further study is warranted to verify the clinical value of plasma cfDNA in HCC. Thus, further research to identify new possible methods to predict the treatment response to TKI-PD-1 is necessary.

Radiomics involves applications of computer vision and artificial intelligence to investigate the hidden characteristics of radiographic images in a quantitative manner^19^. CT images of tumors contain a large amount of useful information that generally cannot be recognized by physicians simply looking at them^20^. The expression of immunotherapy target PD-L1 at the protein level, as well as PD-1 and CTLA4 at the mRNA level, was found by Hectors et al. to be correlated with radiomic features of HCC^21^. Radiomics also showed a powerful ability to predict CD8^+^ T-cell infiltration of HCC in the study of Liao et al.^22^. Therefore, radiomics could be useful in identifying HCC patients who can benefit from immunotherapies. Acquired from routine clinical images, radiomics is a noninvasive, cost-effective method to predict the patient response, and its dynamic changes could be monitored during therapy^23^. Furthermore, the entire three-dimensional tumor landscape is captured by radiomics instead of a small portion of the tumor with spatial heterogeneity as occurs in the biopsy method^24^. Moreover, there is increasing research attention on the importance of the peritumor microenvironment^25^. Results have shown that the fusion rad-score, which consists of features from the peritumoral area, exhibited better performance than the tumor rad-score^26,27^. This indicates that the combination of peritumoral features provides more information on the tumor microenvironment, which can better reflect the biological behavior of the tumor. Therefore, in our study, we examined the entire tumor and its surrounding area, allowing us to collect more information on the tumor and its microenvironment^27,28^.

Machine learning has achieved tremendous success in recent decades. Machine learning is an effective method in the high-throughput era due to the vast amount of data that cannot be directly calculated by our human brains. In 2018, radiomics was demonstrated to be associated with clinical outcomes of cancer patients treated with anti-PD-1 and anti-PD-L1 monotherapy in a retrospective multicohort study^29^ by Sun et al. Recently, Colen et al. found that a radiomics-based signature could predict the response to pembrolizumab in 57 patients with rare types of late-stage cancer with 94.7% accuracy, 97.3% sensitivity, and 90% specificity^30^. Therefore, radiomics-based machine learning may be useful for the development of models to predict TKI-PD-1 efficacy. However, a radiomics signature extracted from baseline CTs of patients with advanced HCC treated with immune checkpoint blockade-based therapy to distinguish patients at risk of progression has not previously been reported. Thus, in this study, following the LASSO selection of 10 radiomic features, ten machine learning algorithms were applied for model construction, with SVM achieving the best performance in both the training and testing cohorts. The preliminary results showed that the SVM method was preferred regarding model construction using radiomics for advanced HCC patients treated with TKI-PD-1, while its efficacy needs to be verified in more studies.

The present study had some inherent limitations. First, whether MRI could provide more information and is more suitable for predicting TKI-PD-1 treatment efficacy needs to be determined. Second, the nature of the study was retrospective with a relatively small sample size of 55 patients because combined TKI-PD-1 therapy (different combinations) has only been applied in recent years. The overfitting problem of machine learning algorithms should be noted, and multicenter studies with large populations and subgroup analyses are warranted in the future. Third, certain tumors could not be delineated due to their infiltrative growth type, and in cases of qualified tumor imaging, only the largest tumor was segmented. Fourth, the performance of the proposed SVM model for prediction was moderate and it needs to be optimized before clinical application. Other machine learning or deep learning methods may be worthwhile in future research. Last, the integration of longitudinal multiomics (genomics, pathomics) data would be clinically significant.

In conclusion, our preliminary study found that radiomics could predict TKI-PD-1 outcomes in patients with advanced HCC. Due to its noninvasive and cost-effective nature, radiomics is a promising approach to pretreatment prediction and decision-making. However, additional prospective studies with a large population obtained from multiple centers are needed to translate the present study into clinical applications.

## Methods

The Ethical Review Committee of Sun Yat-sen University Cancer Center approved the study, and informed consent was obtained from all patients. The study was conducted in accordance with the principles of the Declaration of Helsinki.

### Study design and patient population

**Figure 5:**
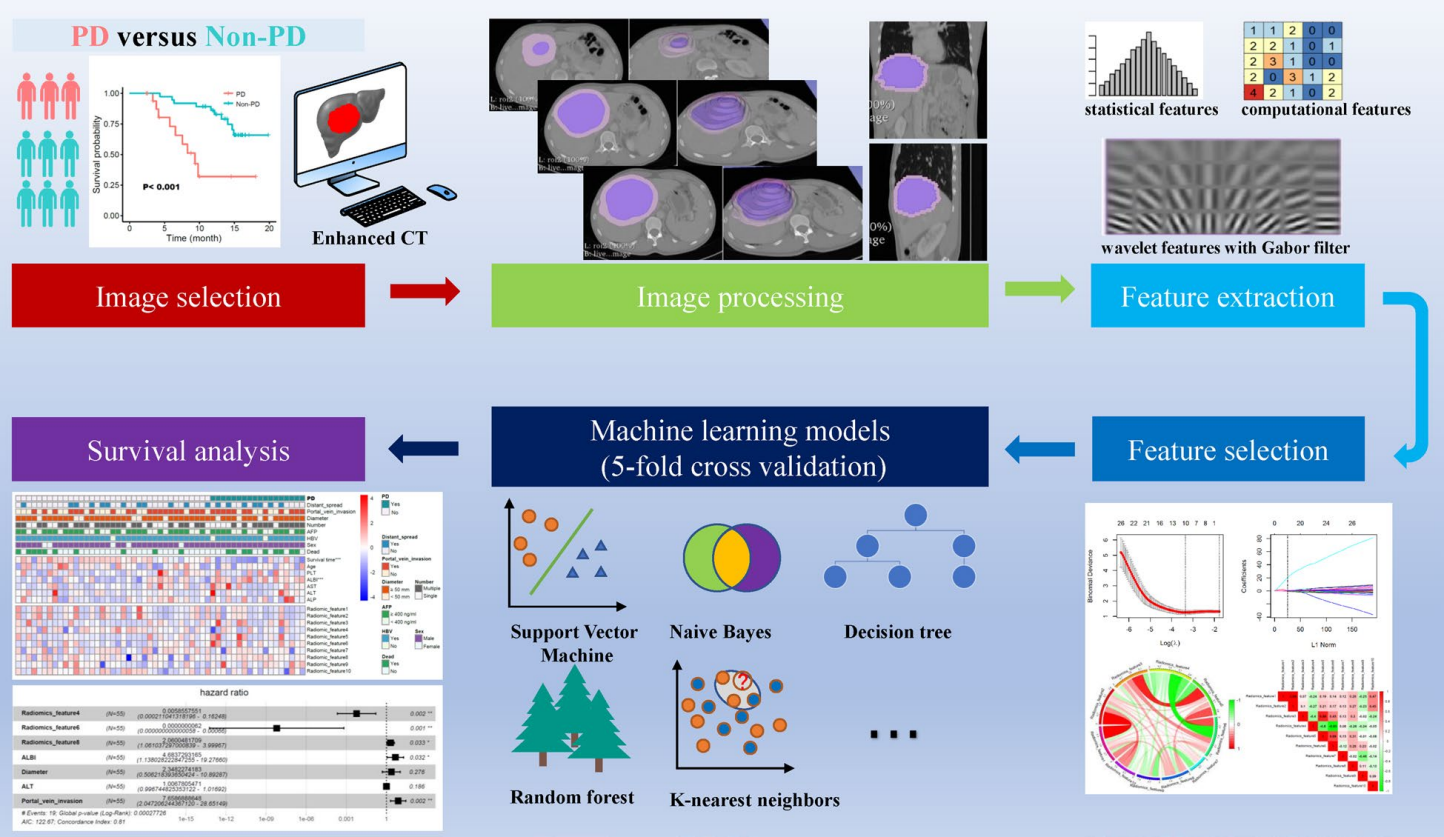
General design of the present study.

The entire study design is shown in Fig. 5. Patients with advanced HCC who received TKIs orally once daily combined with a PD-1 inhibitor given intravenously (TKI-PD-1) from November 2018 to November 2019 were enrolled in this study. Participants were followed from enrollment to the date of death, loss to follow-up, or April 15, 2021.

**Figure 6:**
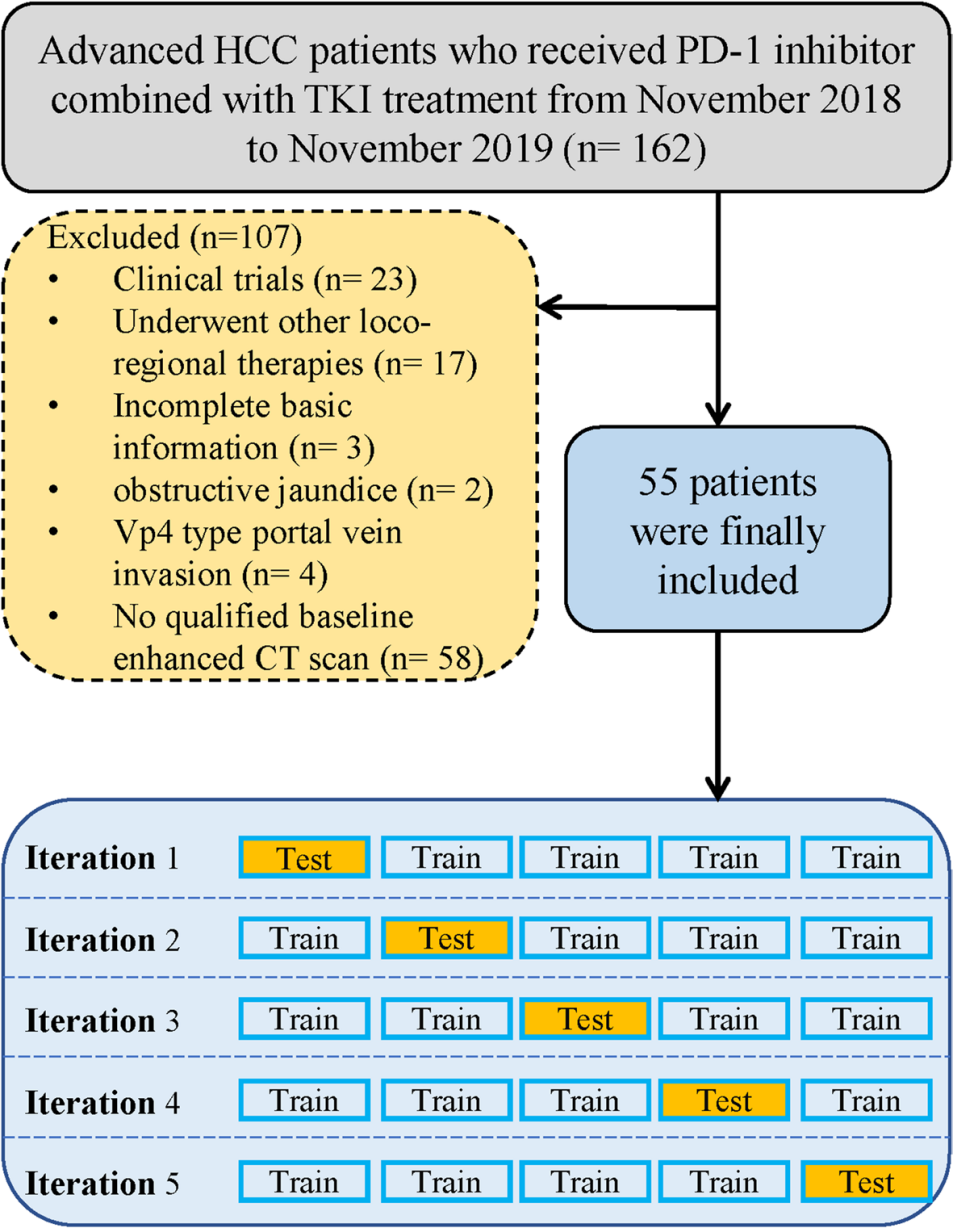
Flowchart of the enrolled patients.

The inclusion and exclusion criteria of patients and treatment protocols were in Supplementary method 1.1 and 1.2. The flowchart of our study is illustrated in Fig. 6.

### Clinical information collection and response evaluation

Data from all enrolled patients were collected, including age, sex, virus infection, PLT, ALBI, ALT, AST, ALP, alpha-fetoprotein (AFP), and survival. The ALBI of each patient was calculated according to the following formula: ALBI = (log_10_ bilirubin × 0.66) − (albumin × 0.085)^31^. In addition, imaging data, including the number and size of tumors, vascular invasion, and extrahepatic metastasis, were collected based on the abdominal CT. All best imaging responses were assessed during treatment on available follow-up images. Progressive disease (PD) was classified into the PD group, while stable disease (SD), partial response (PR), or a complete response (CR) were classified into the non-PD group according to the Response Evaluation Criteria in Solid Tumors V.1.1 guidelines^32^.

### Model construction

Imaging collection and radiomic feature extractions process can be found in Supplementary method 1.3 and 1.4. After the radiomic features were obtained and filtered, ten common machine learning methods were applied to construct possible models, including SVM, NB, Rpart, Ctree, RF, KNN, neuralnet, boosting, bagging, and logistics. Due to the relatively small population, K-fold cross-validation was applied. K-fold cross-validation comprises the following steps: split the data set randomly into 5 folds (11 patients in each fold); construct the model on 4 folds, test the model on the remaining onefold, calculate the error (E) on the observations in the 1 remaining fold; and calculate the average error (AE). The equation is as follows, where k represents the number of folds and Ei indicates the error on the i^th^ iteration:

$$ aE = \left( {1/k} \right)*\mathop \sum \limits_{i = 1}^{k} \left( {Ei} \right) $$

### Statistical analysis

Continuous and categorical variables were compared through Student’s t/Mann–Whitney U test and χ^2^ or Fisher’s exact test. The Spearman method was applied to evaluate the correlations between features. PCA was used to visualize LASSO selected radiomic features in three-dimensional space. The AUC were generated through the R package “pROC”. Radiomics-based machine learning algorithms were developed and compared using the metrics of accuracy, sensitivity, specificity, precision, F1-score, and AUC^33^. Kaplan–Meier curves were plotted to assess OS, and survival differences between two groups were calculated via the log-rank test. A multivariate Cox regression model incorporating clinical and radiomics features was carried out to predict OS. The factors generated in the univariate Cox regression model with a P value less than 0.1 were entered into the multivariate Cox regression model. Decision curve analysis was performed to explore the adding value of radiomic features to clinical variables. For all tests, two-sided *P* < 0.05 was considered statistically significant. All statistical analyses were performed using R software (version 3.6.0).

## Supplementary Information

Supplementary Information.

## Data Availability

Data available on request from the corresponding author.
