# Supplementary material for: Noninvasive imaging-based machine learning algorithm to identify progressive disease in advanced hepatocellular carcinoma receiving second-line systemic therapy
Source: Sci Rep. 2023 Jul 1;13:10690. doi: 10.1038/s41598-023-37862-y (PMC10314898; doi:10.1038/s41598-023-37862-y)
Supplement: Supplementary file 1 — Supplementary Information. [file 41598_2023_37862_MOESM1_ESM.pdf]

Noninvasive imaging-based machine learning algorithm to identify progressive disease in advanced hepatocellular carcinoma receiving second-line systemic therapy

Wei Dong [1#], Ye Ji [2#], Shan Pi [3*], Qi-Feng Chen [4,5,6*]

[1] Department of Medical Oncology, Nanyang Second People's Hospital, Nanyang, China

[2] Department of Medical Oncology, Nanyang Central Hospital, Nanyang, China

[3] Department of Radiology, the Third Affiliated Hospital, Sun Yat-sen University, Guangzhou, Guangdong, China

[4] Department of Medical Imaging and Interventional Radiology, Sun Yat-sen University Cancer Center, Guangzhou, Guangdong, China

[5] State Key Laboratory of Oncology in South China, Guangzhou, Guangdong, China

[6] Collaborative Innovation Center for Cancer Medicine, Guangzhou, Guangdong, China

[#] Contributed equally

*** Correspondence:**
Qi-Feng Chen, Department of Medical Imaging and Interventional Radiology, Sun Yat-sen University Cancer Center, 651 Dongfeng Road East, Guangzhou 510060, China
chenqf25@sysucc.org.cn

Shan Pi, Department of Radiology, the Third Affiliated Hospital, Sun Yat-sen

University, No. 600 Tianhe Road, Guangzhou, 510630, China

pish@mail.sysu.edu.cn

Supplementary method

1.1 Patient population

The patient inclusion criteria were: 1. ≥18 years old; 2. failed first-line treatment; 3. Barcelona Clinic Liver Cancer (BCLC) stage C; 4. Child-Pugh A/B; 5. Eastern Cooperative Oncology Group (ECOG) score ≤2; 6. at least one measurable tumor for evaluation; 7. an expected survival time ≥3 months.

Patients were excluded for any of the following: 1. qualified enhanced CT images prior to therapy were not available; 2. received local-regional treatment; 3. obstructive jaundice; 4. had other malignant tumors; 5. discontinued treatment due to severe adverse events (AEs); or 6. enrolled in any other clinical trials.

Out of the 58 patients who did not have qualified enhanced CT images prior to therapy, 32 had tumors that could not be delineated due to infiltrative growth type. Additionally, 10 patients received an abdomen enhanced MRI and non-enhanced CT scan, while 3 patients had poor image quality and were deemed unsuitable for further analysis. Furthermore, 13 patients had their original DICOM images unavailable due to receiving enhanced CT scans at local hospitals prior to therapy.

1.2 Treatment protocols

Briefly, patients initially received sorafenib (800 mg), lenvatinib (12 mg or 8 mg based on body weight), or apatinib (500 mg) orally daily. Dose reduction was performed based on the severity of toxicities. The toripalimab, pembrolizumab, or

sintilimab was given at 240, 200, or 200 mg, respectively, intravenously on day 1 of each 3-week treatment cycle. The administration of combined therapy was halted after a discussion between patients and physicians when progressive disease occurred. Adverse events were evaluated with CTCAE v. 4.03.

1.3 Imaging collection and radiomic feature extractions

All patients were administered intravenous ionic contrast (Ultravist, Bayer, Germany) with a total dose of 1.5 ml/kg body weight at a speed of 3.0 ml/s. All CT images were obtained using a 64-multidetector CT imaging system (SOMATOM Definition, SIEMENS). Feature extraction was conducted after both the arterial and the portal venous phases of contrast-enhanced CT imaging were obtained. Our region of interest (ROI) included the entire tumor and its surrounding area. First, the entire tumor was subdivided by hand according to the silhouette of the tumor on the CT images slice by slice axially (ROI 1). After that, a 1 cm expanded area along the tumor boundary was automatically generated by 3D Slicer (version 4.11, https://www.slicer.org/) and deemed the peritumoral area (ROI 2). Segmentation was conducted by two radiologists (reader 1 with three years of experience and reader 2 with eleven years of experience) independently to capture the stable features. The silhouette of the largest tumor was delineated when multiple nodules existed. Interobserver reproducibility between the two readers was evaluated by intraclass correlation coefficients (ICCs).

In the present study, the "binWidth" value was set to 25 according to the pyradiomics documentation (https://pyradiomics.readthedocs.io/en/latest/index.html). After the

sizes of the image voxels were resampled at $1 \times 1 \times 1$ mm^3, wavelet filtering was then performed. In total, 2,458 radiomics features were acquired using open-access Pyradiomics, including seven types: first-order features, shape-based (2D and 3D) features, gray level co-occurrence matrix, gray level run length matrix, gray level size zone matrix, neighboring gray tone difference matrix, and gray level dependence matrix.

1.4 Radiomic feature selection

Prior to further analysis, all of the extracted radiomic features were normalized by the z-scores method and filtered by the following steps. After calculating the ICCs of the acquired features between the two readers, we dropped out unstable features (interobserver ICCs < 0.90). Second, among the stable features, candidate features were further determined via the least absolute shrinkage and selection operator (LASSO) approach grouped by nonresponder (PD) and responder (CR + PR + SD).

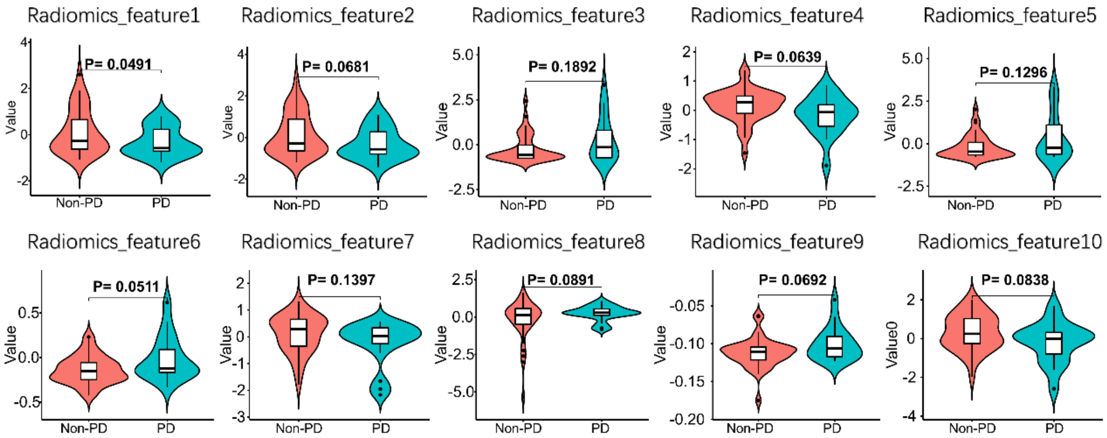

Supplementary Figure 1. Violin plots show distributions of features between PD and non-PD patients, with the horizontal line indicating the median.

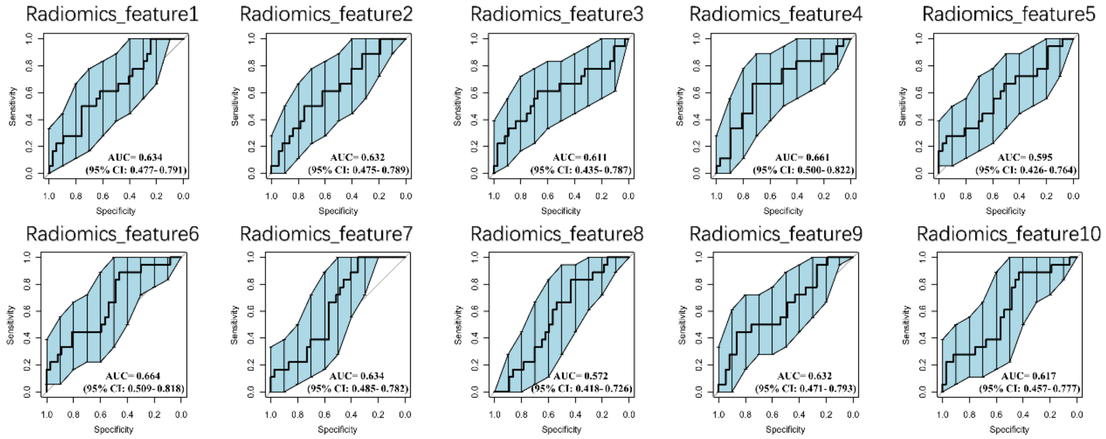

Supplementary Figure 2. Assessment of ten features in predicting PD with the use of receiver operating characteristic (ROC) curves.

Groups	Combinations	N	Death	Median overall survival
**group=1**	camrelizumab plus apatinib	19	7	NA (14.68 - NA)
**group=2**	toripalimab plus lenvatinib	18	6	NA (14.04 - NA)
**group=3**	sintilimab plus lenvatinib	12	4	NA (9.35 - NA)
**group=4**	others	6	2	NA (11.65 - NA)

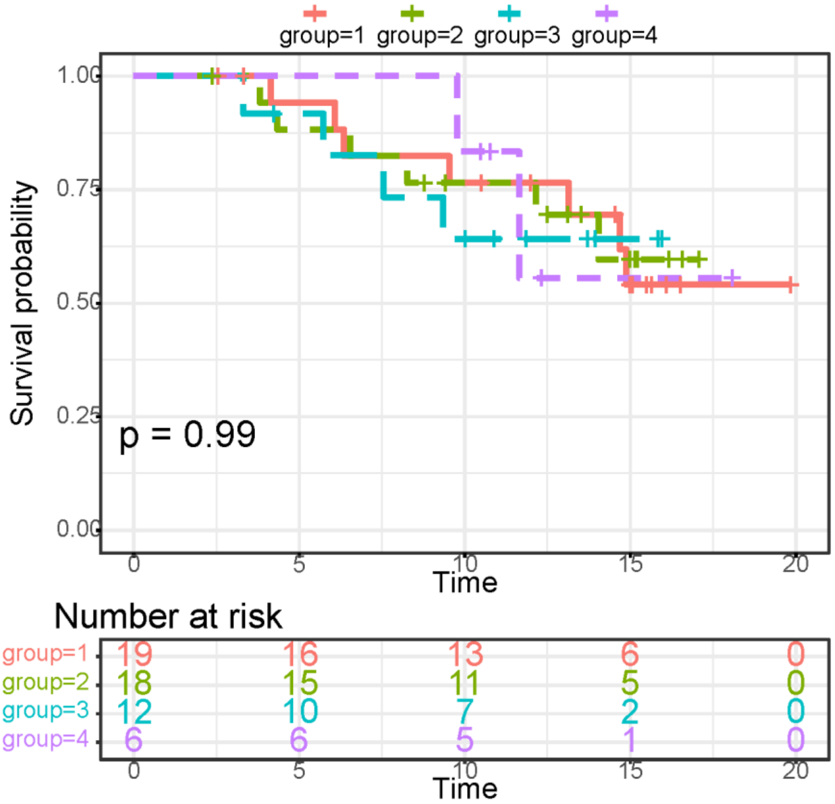

Supplementary Figure 3. Overall survival in subgroups based on the drugs used.

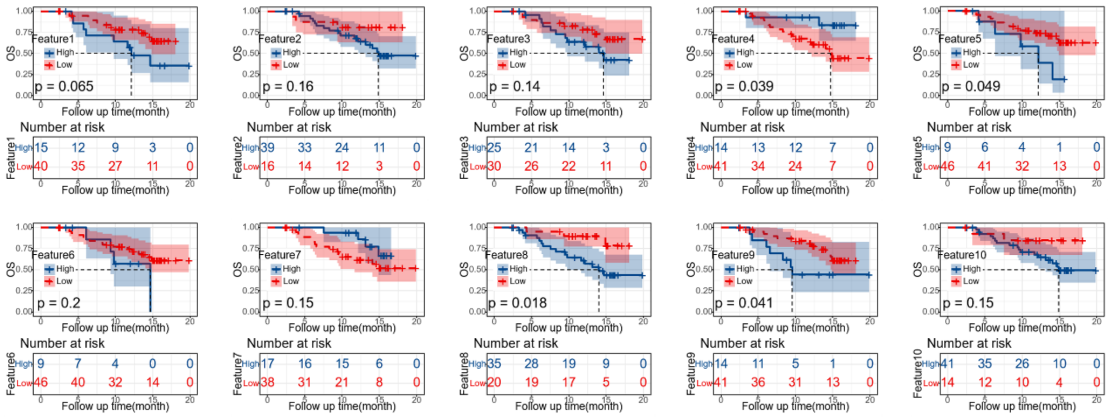

Supplementary Figure 4. Kaplan-Meier curves for OS of the ten radiomic features.

Supplementary Table 1. Ten filtered radiomic features after feature selection.

Radiomic feature order no.	Feature type	Feature name
1	tumor region	wavelet.LHL_glcm_ClusterTendency
2	tumor region	wavelet.LHL_glrlm_GrayLevelVariance
3	tumor region	wavelet.LLL_glszm_LargeAreaHighGrayLevelEmphasis
4	tumor region and the peritumoral region	wavelet.LLH_firstorder_Minimum
5	tumor region and the peritumoral region	wavelet.LLH_glszm_LargeAreaHighGrayLevelEmphasis
6	tumor region and the peritumoral region	wavelet.LLH_glszm_SmallAreaHighGrayLevelEmphasis
7	tumor region and the peritumoral region	wavelet.LHH_firstorder_Median
8	tumor region and the peritumoral region	wavelet.HLL_firstorder_Median
9	tumor region and the peritumoral region	wavelet.HLH_glcm_ClusterShade
10	tumor region and the peritumoral region	wavelet.LLL_glrlm_ShortRunEmphasis

Supplementary Table 2. The results of ten machine learning algorithms using 5-fold iterations.

Group	Mean value				
	Accuracy	F1_score	Precision	Sensitivity	Specificity
**SVM_train**	0.818181818	0.881399815	0.788397664	1	0.436666667
**Bayes_train**	0.736363636	0.799467663	0.816219668	0.783836207	0.636666667
**Rpart_train**	0.804545454	0.854219121	0.855021135	0.858405173	0.693333333
**Ctree_train**	0.672727273	0.804037491	0.672727273	1	0
**RF_train**	1	1	1	1	1
**KNN_train**	0.85	0.894275079	0.8506676	0.945474138	0.65
**Neuralnet_train**	0.95	0.962594973	0.953103448	0.972413793	0.906666666
**Boosting_train**	1	1	1	1	1
**Bagging_train**	0.863636364	0.905841008	0.85485769	0.966810345	0.65
**Logistic_train**	0.768181818	0.829993093	0.816800803	0.843965517	0.606666667
**SVM_test**	0.690909091	0.798976608	0.706363636	0.95	0.2
**Bayes_test**	0.618181818	0.691400851	0.760454545	0.695	0.366666667
**Rpart_test**	0.618181818	0.704449472	0.722698413	0.71	0.433333333
**Ctree_test**	0.672727273	0.79868421	0.672727273	1	0
**RF_test**	0.690909091	0.787822762	0.731883117	0.875	0.3
**KNN_test**	0.672727272	0.763968254	0.748095238	0.825	0.433333333
**Neuralnet_test**	0.690909091	0.753932179	0.781666667	0.745	0.566666667
**Boosting_test**	0.672727272	0.771278195	0.727994228	0.85	0.333333333
**Bagging_test**	0.6	0.736321195	0.657777778	0.85	0.066666667
**Logistic_test**	0.636363636	0.741460268	0.690295815	0.81	0.233333333
